# The Health of London

**Published:** 1904-01-23

**Authors:** 


### The Hospital.

A JOURNAL OF

The Medical Sciences and Hospital Administration.

VOL. XXXV.—No. 904. JANUARY 23, 1904.

### THE HEALTH OF LONDON.

THE very valuable report and appendices annually presented to the London County Council by its zealous Medical Officer of Health, Mr. Shirley Murphy, belong to that too numerous class of documents which, for one reason or another, are not given to the public until the time to which they relate has passed away, and until the interest originally excited by the events which they record has been superseded by that arising from more recent occurrences. In the beginning of the year 1904 we are presented with an elaborate review of the health of the Metropolis as it was in the course of 1902; and our attention is strongly directed to legislative or administrative shortcomings which may have been remedied before the story of their existence is told. We have congratulations upon the fall of the annual death-rate of the Metropolis to 17·2 per 1,000 living, congratulations which lose much of their effect when compared with the death-rate of 13·5 for the third quarter of 1903, as arrived at by exclusion of the mortality occurring in London to persons ordinarily resident in the country, and who had come to the metropolis for operative or other treatment of dangerous disease. But, apart from these comparatively belated portions of the report, we have also a record of continuous progress in the enforcement of judicious sanitary control over those sections of the population for which such control is most urgently required, as well as of unslumbering vigilance in the discharge of duties of the highest importance to all residents in the metropolis. The only difficulty in dealing with these aspects of the volume is that which depends upon an *embarras de richesse*, upon the very number and importance of the circumstances to which they relate.

The first place may perhaps be given to the story of the consequences of one of the most unfortunate of modern quasi-scientific pronouncements, that by which Professor Koch succeeded for a time in casting doubt upon the communicability of bovine tuberculosis to mankind. An outbreak of scarlet fever which occurred in East London in 1901, and which was traced to infected milk from a farm in Staffordshire, directed the attention of the County Council to the fact that there are no powers under the existing law to exclude an infected milk supply from London as a whole, and that the only machinery which can be put in force for the protection of the twenty-nine sanitary districts into which the county is divided is of a kind which involves serious delay. In these circumstances the council, acting under the advice of its Sanitary Committee and of its medical officer of health, decided to apply to Parliament, in its General Powers Bill of 1902, for further powers on this subject, and also in relation to tuberculous milk, but has to announce its regret that the legislation asked for was not granted, although similar powers have been given to the local authorities of a number of districts outside London. The report adds that the pronouncement of Professor Koch, to the effect that there is little probability of tuberculosis being communicated from animals to man, was, "of course," quoted against the council's proposals, and the Sanitary Committee is, therefore, "interested to observe" that recent experiments by other authorities have "not tended to support" the Professor's conclusions. It is a little too much that the health of London, in the twentieth century of the Christian era, should be even partly dependent upon hypotheses "made in Germany;" and we can only wish our legislators more sense than on this occasion they appear to have displayed. Nothing could show more convincingly the urgent need which exists for a responsible Minister of Health than the fact that a perfectly proper and reasonable request, founded upon clear evidence and supported by abundant precedent, has been passed over or refused for no apparent reason. The Local Government Board, even although its President is actuated by the best possible intentions, and has access to the most judicious and most skilled advice, is far too much hampered by administrative duties of various kinds to have either sufficient leisure for the study of questions relating to the sanitary requirements of the population, or sufficient authority in connection with them, to be able to press them upon the attention of a reluctant and frequently ignorant legislature.

The position of oysters and other shellfish in relation to enteric fever is another subject which has received the attention of the Council, and it is recorded that the Local Government Board has been urged to seek such an amendment of the law as would prohibit, under heavy penalties, either the laying down of edible forms of shellfish in sewagepolluted creeks or other dangerous localities, or the sale of shellfish which had been so laid down for human consumption, as well as the future pollution of layings and beds which are at present in a condition of safety. We need hardly say that this judicious counsel has been barren of result, and that the whole question has been suffered to drift while the lately issued report of the Royal Commission upon Sewage was being waited for. Now that this has appeared we shall still have to wait, even if its recommendations are adopted by the Government, until the projected "Rivers Board" is constituted and brought into operation; and in the meanwhile must derive what consolation we can from the information that the *bacillus coli communis* is too frequently found in oysters, in numbers ranging from 10 to 10,000, for its presence to afford any trustworthy indication that they have been fed in sewage polluted water, or any valid reason for avoiding them as articles of food. The Commission even suggests the possibility, at some future time, of the adoption of a numerical test of safety or indication of peril. An oyster will perhaps be permitted to possess a certain (or at present an uncertain) number of colon bacilli without being rejected for the purposes of the table, but any considerable excess may render him a suspicious character, and place him upon the *Index Expurgatorius* of the kitchen. We are inclined to take a shorter way, and, in the absence of the effective legislation to say to him:

"Cassio, I love thee;

But never more be officer of mine."

